# Comparison of the Effectiveness of Platelet-Rich Plasma Injection Versus Plantar-Specific Calf Stretching Exercises in Patients With Plantar Fasciitis: A Randomized Controlled Trial

**DOI:** 10.7759/cureus.67992

**Published:** 2024-08-28

**Authors:** Akhilesh S Khobragade, Devashis Barick, Kunal Parmar, Virendra E Patil, Sarang Rokade, Suhas Waghe

**Affiliations:** 1 Orthopaedics and Traumatology, N. K. P. Salve Institute of Medical Sciences & Research Centre and Lata Mangeshkar Hospital, Nagpur, IND; 2 Orthopaedics, N. K. P. Salve Institute of Medical Sciences & Research Centre and Lata Mangeshkar Hospital, Nagpur, IND

**Keywords:** calf stretching exercises, the visual analogue scale, pes planus, plantar fascitis, platelet rich plasma

## Abstract

Background

Plantar fasciitis is a common foot condition with multifactorial etiology. It is the most frequent cause of heel pain and has been categorized as an overuse syndrome. A clinical examination and history are crucial for diagnosis. There are several different forms of treatment available, two of which are frequently used: physical therapy and steroid injections. Recent research on platelet-rich plasma (PRP) has demonstrated encouraging outcomes and fewer side effects when compared to steroid injections.

Methods

A randomized controlled trial was conducted and randomization was done of indoor patients into two groups. Group 1, ending with odd numbers, was given PRP injections, and Group 2, ending with even numbers, was advised plantar-specific calf stretching exercises. Visual analog scale (VAS) scores were evaluated before and after the intervention and follow-up was done on the second, sixth, and 12^th^ weeks.

Results

Comparing the VAS scores between the two groups, we found that in the pre-intervention phase, the VAS score of Group 1 was 5.4±0.56 and that of Group 2 was 5.4±0.59. In the post-intervention phase, the VAS score in Group 1 was 4.6±0.89, while in Group 2 it was 5.2±0.62. In the second week after intervention, the VAS score was observed to be 3.3±0.97 in Group 1, while in Group 2, it was 3.3±0.80. After the sixth week of intervention, the observed VAS score was 2.7±0.78, while in Group 2 it was 2.9±0.82. The mean VAS score after 12weeks of intervention was observed to be 2.3±0.91 in Group 1, while in Group 2, it was 2.2±0.80.

Conclusion

PRP injections and plantar-specific calf stretching exercises are equally effective in providing pain relief in plantar fasciitis. PRP injections have complications and problems which have been discussed. Exercises are devoid of such complications. No recurrences occurred in the exercise group and four cases had recurrence in the PRP group.

## Introduction

Plantar fasciitis is a common foot condition and has been classified as a syndrome resulting from repeated trauma at its origin on the calcaneus [1]. It is considered an overuse injury ensuing from external and internal factors [2]. External factors embody training errors coaching on unyielding surfaces and improper or faulty worn footwear [3]. Internal factors include obesity, foot structure, reduced plantar flexion strength, stiffening of plantar flexor muscles, and torsional mal-alignment of the lower extremity.

Flexion of the toes, specifically the great toe, activates a lifting device-like mechanism known as the windlass mechanism that passively tenses the plantar fascia and elevates the medial longitudinal arch [4]. It is now taken into account that plantar fasciitis is a self-limited condition and the symptoms have been shown to resolve in 80-90% of cases in a duration of 10 months [5]. The most common associated symptom is pain and discomfort in the heel region which aggravates on weight bearing after a period of non-weight bearing [6]. Patients describe it as an excruciating pain present when waking up from bed in the morning and subsiding within 35-45 minutes or throughout the course of the day [1].

There’s no single treatment for plantar fasciitis and the condition often responds to a wide array of conservative therapies. Modalities include rest, physical therapy, deep X-ray therapy [7], nonsteroidal anti-inflammatory drugs (NSAIDs), steroid injections, foot pad support, shoe modifications, arch and custom foot orthoses [8], and night splints [9]. Of these interventions, the plantar-specific calf stretch exercise plays a major role in the treatment by stretching and strengthening programs. Increasing the flexibility of the calf muscles is necessary [10].

Frequently used stretching techniques are wall stretches and curb, stepper, or stair stretches. Stretching exercises, which are considered central to most treatment protocols, are assessed either on their own or for their long-term benefits [11].

In recent times, platelet-rich plasma (PRP) has shown promising results because when compared to steroids they are not associated with complications such as repeated local injections [12]. PRP has proved to be an excellent autologous biological blood-derived product. When applied to various types of tissues, it releases a substantial quantity of platelet-derived growth factors, which support bone regeneration, wound healing, and tendon repair [12]. This has led to growing enthusiasm for the use of PRP as it provides significant pain relief and functional improvement and thus results seem comparable or superior to steroid injection. Hence, the present study was conducted to assess the effectiveness of PRP in causing pain relief in patients with plantar fasciitis compared to patients undergoing plantar-specific calf stretching exercises.

## Materials and methods

This was a prospective randomized analytical comparative trial conducted for three years at N. K. P. Salve Institute of Medical Sciences & Research Centre and Lata Mangeshkar Hospital, Nagpur, Maharastra, India. The study has been registered at the Clinical Trials Registry-India (CTRI) (registration number: REF/2022/07/055999). The study was approved by the Institutional Ethics Committee of N. K. P. Salve Institute of Medical Sciences & Research Centre and Lata Mangeshkar Hospital (approval number: 62/2017).

A total of 66 patients, who underwent treatment for plantar fasciitis (passive stretch exercises or PRP injection) at a tertiary care center, were recruited for the study. Randomization was done based on the inpatient department (IPD) numbers ending in odd or even numbers. Patients were randomized into one of the two groups: Those with an odd number were allotted Group 1 (n=33), the PRP injection group, and those with an even number were allotted Group 2 (n=33), the plantar-specific calf stretching exercise group.

Patients in Group 1 were given PRP injections. About 15 ml of whole blood was collected from the unaffected arm into a 20 ml syringe that contained 5 ml sodium citrate. A peripheral complete blood count was also collected at the time of the initial blood draw. The blood was then prepared with reference to the manual according to GPS® III Platelet Concentration System (Zimmer Biomet Holdings, Inc., Warsaw, Indiana, United States) processing instructions. The sample was centrifuged for five minutes at 1500 rpm. Then the buffy layer coat was separated and the sample was again centrifuged for five minutes. The PRP obtained was injected into the heel of the patient at the site of maximum tenderness.

Patients in Group 2 were given a demonstration of the exercises such as wall leaning, ball rolling, and towel curls exercises. Patients were told to perform these exercises and follow-up was taken after two weeks, four weeks, and three months of exercises using visual analog scale (VAS) scoring of the foot.

Sample size

We calculated the sample size with the help of OpenEpi (www.OpenEpi.com) and with the following considerations: two-side confidence level, 95%; power of study, 80%; ratio of exposed to unexposed, 1:1; percentage of unexposed with outcome, 92%; percentage of exposed with outcome, 64.3%. After calculating the sample size and the loss to follow-up as 10%, we rounded out the value. The sample size was calculated to be 66 patients including both groups, with 33 patients in each.

Inclusion and exclusion criteria

The inclusion criteria were: patients with pain in the heel region for more than one month, aged more than 18 years, patients who have not got relief with pharmacotherapy, and VAS score in the morning >5.

Patients who had undergone other modalities of treatment such as steroid, bracing, extracorporeal shock wave therapy, who have undergone foot surgery, who had an anatomical deformity of the foot or any previous surgery, patients with a history of diabetes mellitus and neuropathy-related heel pain, and patient who did not have the ability to understand informed consent were excluded from the study.

Process

The patient’s general demographic data, and previous clinical, medical, and treatment history were enquired about at the time of the initial visit. Additionally, BMI was measured. Patients were called for a follow-up visit on the second, sixth, and 12th week by the principal investigator, and their VAS score was noted. Outcome measures included plantar heel pain, which was measured as per VAS scores. The VAS recorded the patient-reported pain on a scale of 0-10 where 0 is pain-free and 10 is the worst pain imaginable.

The treatment was defined as successful if the pain reduction after six months was over 25%. If the patient was lost to follow-up, the last measurement was carried forward. If the patient obtained a different treatment, the subject was classified as an unsuccessful post-procedural follow-up.

Data Management

A master chart containing two parts, one for the PRP group and one for the plantar-specific calf stretching exercise was made which contained all the critical information about the patients recruited for the study.

Statistical analysis

The observations of this study along with the case record forms and master chart were taken to the Board of Research Studies-approved statistician at our center. A p-value of < 0.05 was considered for our study to be significant and calculations were done with IBM SPSS Statistics for Windows, Version 26.0 (Released 2019; IBM Corp., Armonk, New York, United States) and data was analyzed. The analyzed data was presented in tables and charts. Mean VAS scores before treatment, immediately after treatment, after two weeks, six weeks, and 12 weeks were calculated and respectively t score value and p-value were compared.

## Results

The data of 66 patients was analyzed to compare the effectiveness of plantar-specific calf stretching exercises versus PRP injections in chronic plantar fasciitis by comparing the VAS score pre-and post-treatment at intervals of two weeks, six weeks, and 12 weeks.

**Figure 1:**
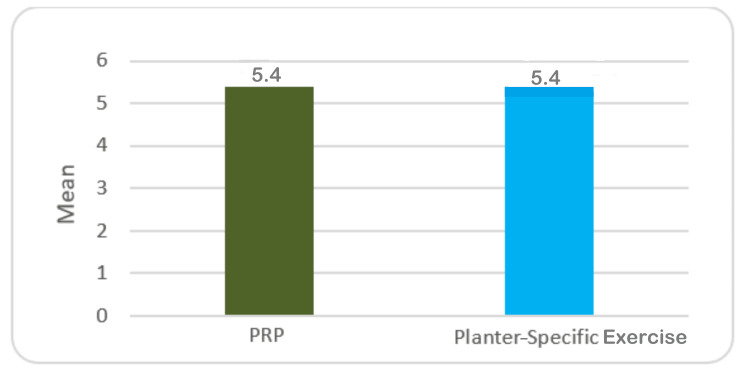
Mean VAS score before treatment with PRP and planter-specific exercises VAS: visual analog scale; PRP: platelet-rich plasma

Table 1 shows results pertaining to the VAS score of plantar fasciitis patients before treatment with PRP and plantar-specific calf stretching exercise. The mean VAS score of patients belonging to the PRP group was 5.4±0.56, while the exercise group differed very slightly with a mean VAS score of 5.4±0.59. There was no statistically significant difference (Table 1, Figure 1).

**Table 1:** VAS score of plantar fasciitis patients before treatment with PRP and plantar-specific calf stretching exercise. SD: standard deviation; SE: standard error; MD: mean deviation; VAS: visual analog scale; PRP: platelet-rich plasma

Groups	Mean	SD	SE	Minimum	Maximum	MD	t ratio	p-value
PRP group (n=33)	5.4	0.56	0.10	4	7	0.03	0.239	0.813
Exercise group (n=33)	5.4	0.59	0.10	5	7			

**Figure 2:**
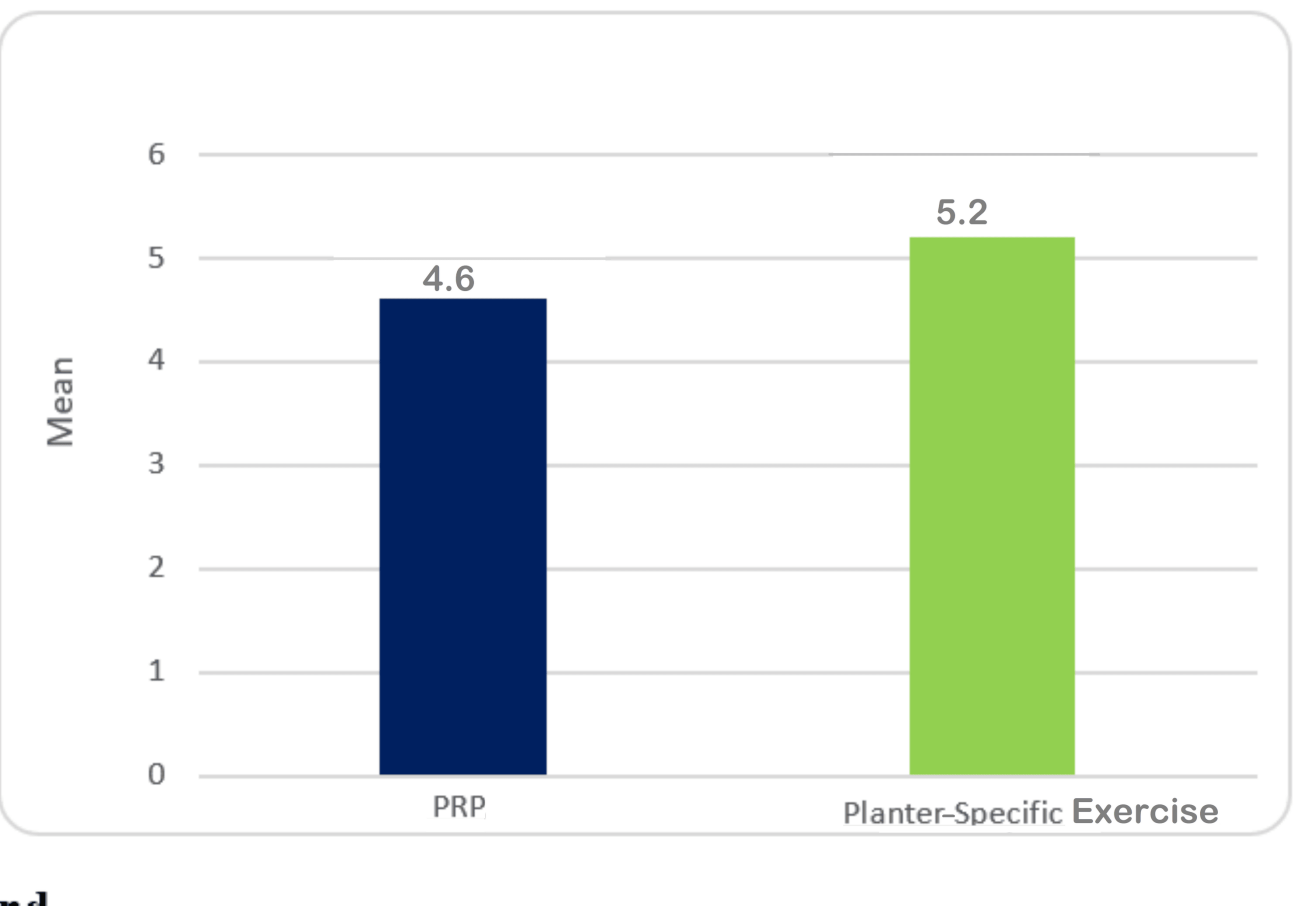
Mean VAS score of plantar fasciitis patients immediately after treatment with PRP and plantar-specific calf stretching exercises VAS: visual analog scale; PRP: platelet-rich plasma

Table 2 shows that the mean VAS score of patients belonging to the PRP group was 4.6±0.89, while that of the exercise group was 5.2±0.62. The comparative assessment showed that there is a statistically significant (p < 0.05) difference in the VAS score just after the treatment (Table 2, Figure 2).

**Table 2:** VAS score of plantar fasciitis patients just after the treatment with PRP and plantars-specific calf stretching exercises. SD: standard deviation; SE: standard error; MD: mean deviation; VAS: visual analog scale; PRP: platelet-rich plasma

Groups	Mean	SD	SE	Minimum	Maximum	MD	t ratio	p-value
PRP group (n=33)	4.6	0.89	0.17	3	6	-0.59	-2.842	.009
Exercise group (n=33)	5.2	0.62	0.12	4	7			

**Figure 3:**
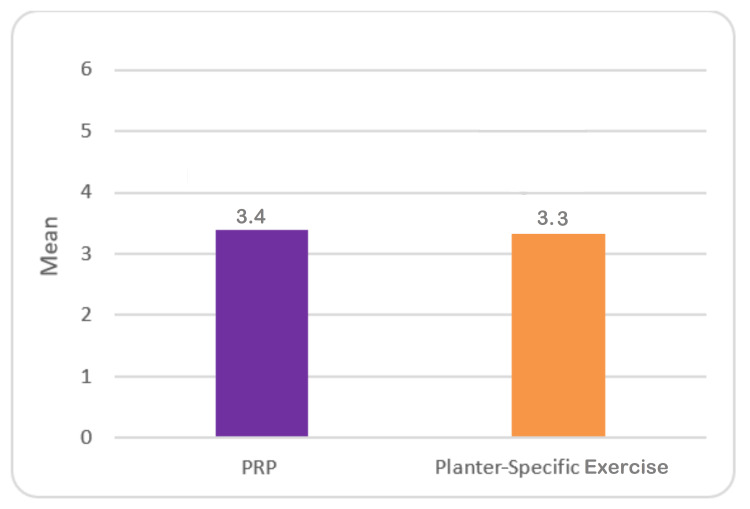
Mean VAS score of plantar facilities patients after two weeks of treatment with PRP and plantar-specific calf stretching exercise VAS: visual analog scale; PRP: platelet-rich plasma

Table 3 shows that it was observed that the mean VAS score of patients belonging to the PRP group was 3.3±0.97, while the exercise group was 3.3±0.80. The comparative assessment indicated that there was no statistically noticeable difference after two weeks (Table 3, Figure 3).

**Table 3:** VAS score of plantar facilities patients after two weeks of treatment with PRP and plantar-specific calf stretching exercise. SD: standard deviation; SE: standard error; MD: mean deviation; VAS: visual analog scale; PRP: platelet-rich plasma

Groups	Mean	SD	SE	Minimum	Maximum	MD	t ratio	p-value
PRP group (n=33)	3.4	0.97	0.19	2	7	-0.04	-.166	.869
Exercise group (n=33)	3.3	0.80	0.15	2	5			

**Figure 4:**
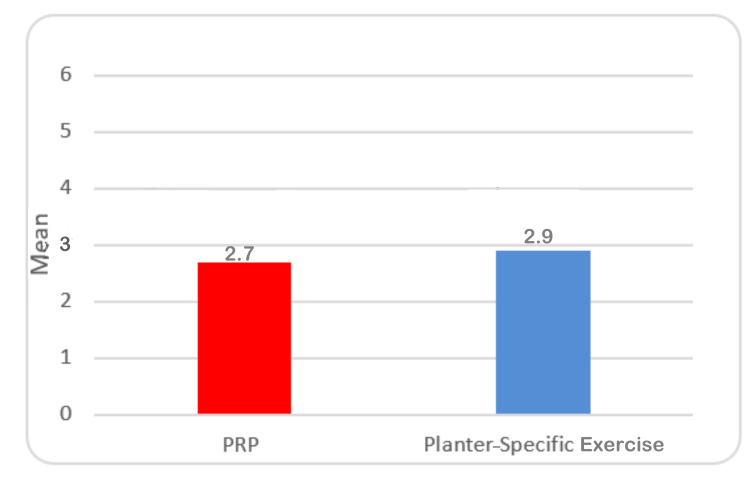
Mean VAS score of plantar fasciitis patients after six weeks of treatment with PRP and plantar-specific calf stretching exercise VAS: visual analog scale; PRP: platelet-rich plasma

Table 4 shows that it was observed that the mean VAS score of patients belonging to the PRP group was 2.7±0.78, while that of the exercise group was 2.9±0.82. The comparative assessment indicated that there was no statistically noticeable difference after six weeks (Table 4, Figure 4).

**Table 4:** VAS score of plantar fasciitis patients after six weeks of treatment with PRP and plantar-specific calf stretching exercise SD: standard deviation; SE: standard error; MD: mean deviation; VAS: visual analog scale; PRP: platelet-rich plasma

Groups	Mean	SD	SE	Minimum	Maximum	MD	t ratio	p-value
PRP group (n=33)	2.7	0.78	0.15	1	4	-0.19	-1.095	.284
Exercise group (n=33)	2.9	0.82	0.16	1	4			

**Figure 5:**
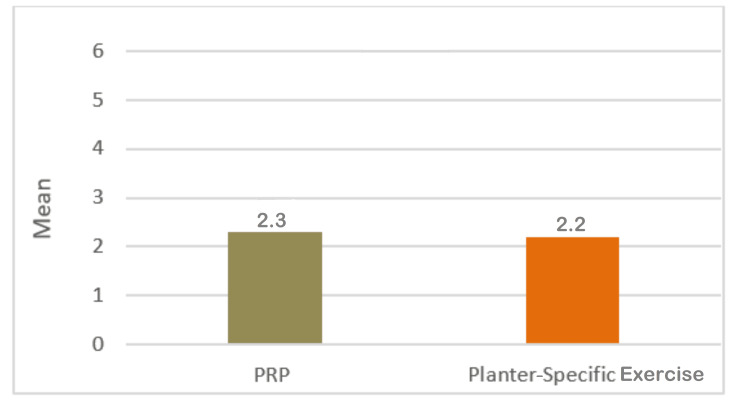
Mean VAS score of plantar fasciitis patients after 12 weeks of treatment with PRP and plantar-specific calf stretching exercise SD: standard deviation; SE: standard error; MD: mean deviation; VAS: visual analog scale; PRP: platelet-rich plasma

Table 5 shows that it was observed that the mean VAS score of patients belonging to the PRP group was 2.3±0.91, while that of patients belonging to the exercise group was 2.2±0.80. The comparative assessment indicated that there is no statistically noticeable difference after 12 weeks of treatment (Table 5, Figure 5)

**Table 5:** VAS score of plantar fasciitis patients after 12 weeks of treatment with PRP and plantar-specific calf stretching exercise SD: standard deviation; SE: standard error; MD: mean deviation; VAS: visual analog scale; PRP: platelet-rich plasma

Groups	Mean	SD	SE	Minimum	Maximum	MD	t ratio	p-value
PRP group (n=33)	2.3	0.91	0.18	0	4	0.07	.311	.758
Exercise group (n=33)	2.2 ±	0.80	0.15	0	4			

Table 6 shows the results of all pair-wise comparisons of VAS scores of the PRP group patients obtained at different time intervals using Tukey's honestly significant difference (HSD). The VAS scores obtained after six and 12 weeks indicate that there is no significant difference in these two scores; however, they are distinctly different than the scores obtained at other time intervals (Table 6).

**Table 6:** Comparative assessment of the VAS score of patients belonging to the PRP group VAS: visual analog scale; PRP: platelet-rich plasma

Duration	Number	Subset for alpha = 0.05			
		1	2	3	4
After 12 Weeks	31	2.2903			
After 6 Weeks	31	2.6129			
After 2 Weeks	31		3.4516		
Just After	31			4.5806	
Before	31				5.3871

The VAS scores of plantar-specific calf stretching exercises were taken before and immediately after exercise, and after two weeks, six weeks, and 12 weeks of exercise. Subsets 1 and 2 in Table 7 indicate that these VAS scores were considerably different from each other.

**Table 7:** Comparative assessment of the VAS score at different time intervals of patients belonging to exercise group. VAS: visual analog scale

Duration	Number	Subset for alpha = 0.05			
		1	2	3	4
After 12 Weeks	28	2.2143			
After 6 Weeks	28		2.8571		
After 2 Weeks	28			3.3929	
Just After	27				5.1852
Before	30				5.3667

## Discussion

Plantar fasciitis is a common overuse syndrome of the foot resulting due to repeated trauma at the origin of the plantar fascia. It is challenging for many clinicians to treat with success [2]. Combination therapy has been proven to be most effective in causing pain relief in patients with plantar fasciitis [13]. Though many authors have claimed individually significant relief in symptoms in both exercise and PRP treatments, no study has yet compared them together to the best of our knowledge. The results of the current study demonstrated that there is no noticeable difference in the reduction of VAS scores when comparing plantar-specific calf stretching exercise and PRP. Early diagnosis and treatment have better patient-related outcomes in terms of duration of treatment [14].

Patients with acute plantar fasciitis respond better to conservative management. However, chronic plantar fasciitis may have some permanent changes. such as degenerated or hypertrophied bundles and may not respond to conservative management [15].

The mean duration of symptoms prior to treatment of the PRP group and plantar-specific calf stretching exercise group were 69.19 and 68.61 days, respectively. Therefore, we can say both groups had an almost similar duration of symptoms and there was not any difference in the duration and chronicity of the disease in one specific group. In our study, in the pre-intervention phase, the mean VAS score of the PRP group was 5.4±0.56 and that in the exercise group was 5.4±0.59. Another study done by Engkananuwat et al. in 2018 comparing the effectiveness of stretching exercises in plantar fasciitis reported a pre-treatment mean VAS score of 4.4 [16].

In the post-intervention phase, we observed that the mean VAS score in the PRP group was 4.6±0.89, while in the Exercise group, it was 5.2±0.62. The comparative assessment showed that there is a statistically significant difference (P < 0.05) in the VAS score in both groups, with the PRP group showing a lower VAS score.

The mean VAS score in the second week after the intervention was observed to be 3.3±0.97 in the PRP group, while in the Exercise group, it was 3.3±0.80. Again, no significant difference was observed. However, the PRP group showed a lower VAS score. In a meta-analysis of eight treatment modalities by Li et al., they reported that on the one-month follow-up, the VAS score for PRP injections was 5.34 [17]. The study done by Engkananuwat et al. showed that at the end of the third month of follow-up, the reported VAS score was 2.0 after exercises [18]. In the meta-analysis done by Li et al., it was found that on the third-month follow-up, the VAS score for PRP injections was 2.78 [17].

Engkananuwat et al. reported a VAS score of 3.0 after exercises at the fourth-month follow-up [16]. In the present study, the mean VAS score after the sixth week of intervention was observed to be 2.7±0.78, while that of patients belonging to the Exercise group was 2.9±0.82. A similar trend was observed with the PRP group showing a lower VAS score.

In the current study, the mean VAS score after the 12th week of intervention was observed to be 2.3±0.91, while that of the Exercise group was 2.2±0.80. The comparative assessment showed a similar trend again, with the PRP group showing a lower VAS score.

According to the literature, many studies have claimed the superiority of PRP injections compared to steroid injections in terms of reducing the symptoms related to plantar fasciitis. Also, PRP is devoid of complications such as rupture of plantar fascia, fat pad atrophy, and osteomyelitis of the calcaneus, which are commonly associated with corticosteroid injections [18]. It was our endeavour to compare PRP versus plantar-specific calf stretching exercises and our study shows no statistically noticeable difference in the VAS scores between the two modalities. However, on long-term follow-up of patients, there were four incidences of recurrence which belonged to the PRP group and none from the exercise group. So, we consider that the exercise group did well in the long term and proved to be superior as compared to PRP.

Limitations

A limitation of our study was that it was a single-center study. The sample size was also small. We recommend conducting this study at multiple centers with an increased sample size to strengthen and further validate our result, thus reducing the chances of errors and bias.

## Conclusions

PRP injections and plantar-specific calf stretching exercises are equally effective in providing pain relief in plantar fasciitis. PRP injections may have their own set of complications and problems. In our study, none of the patients had a recurrence of symptoms in the exercise group and there were recurrences of symptoms in four cases that belonged to the PRP group. Considering the results and outcomes in both groups of patients we are inclined to deduce that plantar-specific calf stretching exercises are a more reliable, efficient, and effective therapy, bereft of any complications and a cheaper method of providing sustainable relief of pain in patients with plantar fasciitis as compared to PRP injections.

## References

[1] Cornwall MW, McPoil TG (1999). Plantar fasciitis: etiology and treatment. J Orthop Sports Phys Ther.

[2] Krivickas LS (1997). Anatomical factors associated with overuse sports injuries. Sports Med.

[3] Nunn NR, Dyas JW, Dodd IP (1997). Repetitive strain injury to the foot in elite women kendoka. Br J Sports Med.

[4] League AC (2008). Current concepts review: plantar fasciitis. Foot Ankle Int.

[5] Davis PF, Severud E, Baxter DE (1994). Painful heel syndrome: results of nonoperative treatment. Foot Ankle Int.

[6] Kosmahl EM, Kosmahl HE (1987). Painful plantar heel, plantar fasciitis, and calcaneal spur: etiology and treatment. J Orthop Sports Phys Ther.

[7] RO GK (1955). The painful heel. Br Med J.

[8] Seegenschmiedt MH, Keilholz L, Katalinic A, Stecken A, Sauer R (1996). Heel spur: radiation therapy for refractory pain--results with three treatment concepts. Radiology.

[9] Wapner KL, Sharkey PF (1991). The use of night splints for treatment of recalcitrant plantar fasciitis. Foot Ankle.

[10] Young CC, Rutherford DS, Niedfeldt MW (2001). Treatment of plantar fasciitis. Am Fam Physician.

[11] Rompe JD, Cacchio A, Weil L Jr (2010). Plantar fascia-specific stretching versus radial shock-wave therapy as initial treatment of plantar fasciopathy. J Bone Joint Surg Am.

[12] Bacevich BM, Smith RD, Reihl AM, Mazzocca AD, Hutchinson ID (2024). Advances with platelet-rich plasma for bone healing. Biologics.

[13] Steinmetz M (1999). Treatment choices for plantar fasciitis. Am Fam Physician.

[14] Gill LH, Kiebzak GM (1996). Outcome of nonsurgical treatment for plantar fasciitis. Foot Ankle Int.

[15] Alghadir A (2006). Conservative treatment of plantar fasciitis with dorsiflexion night splints and medial arch supports: a prospective randomized study [Thesis]. Doctoral Dissertation, University of Pittsburgh.

[16] Engkananuwat P, Kanlayanaphotporn R, Purepong N (2018). Effectiveness of the simultaneous stretching of the Achilles tendon and plantar fascia in individuals with plantar fasciitis. Foot Ankle Int.

[17] Li H, Lv H, Lin T (2018). Comparison of efficacy of eight treatments for plantar fasciitis: a network meta-analysis. J Cell Physiol.

[18] Kennedy JC, Willis RB (1976). The effects of local steroid injections on tendons: a biomechanical and microscopic correlative study. Am J Sports Med.

